# Prenatal vitamin D status and long-term neurocognitive outcomes: building on an emerging evidence base

**DOI:** 10.1038/s41390-025-04476-1

**Published:** 2025-10-08

**Authors:** Carol L. Wagner, Bruce W. Hollis

**Affiliations:** 1 College of Medicine, Pediatrics, Medical University of South Carolina, Charleston, SC USA; 2 Division of Neonatology, Shawn Jenkins Children’s Hospital, Medical University of South Carolina, Charleston, SC USA; 3 Pediatrics, Medical University of South Carolina, Charleston, SC USA

Vitamin D sufficiency during pregnancy has long been recognized for its importance in fetal bone development. Still, a growing body of evidence is shedding light on its broader role in neurodevelopment.^1–6^ The recently published manuscript by Valera-Gran et al. in *Pediatric Research* contributes meaningfully to this evolving field.^7^ Drawing on the ECLIPSES cohort, a prospective study of pregnant women and their offspring in Catalonia, Spain, the authors present compelling evidence that higher maternal 25-hydroxyvitamin D [25(OH)D] concentrations during pregnancy are positively associated with improved neurocognitive outcomes in children at four years of age. These findings reinforce earlier reports and offer new insights into the importance of trimester-specific maternal vitamin D status on child development.

The ECLIPSES study followed 289 mother-child pairs, with measurement of maternal 25(OH)D concentrations during the first and third trimesters and assessment of neurodevelopment at age four years using a comprehensive battery of validated instruments: the Wechsler Preschool and Primary Scale of Intelligence (WPPSI-IV; which assesses a child’s intellectual development across various areas, including verbal comprehension, visual-spatial reasoning, fluid reasoning, working memory, and processing speed in preschool children from 2 years 6 months up to 7 years 7 months), the Developmental NEuroPSYchological Assessment, Second Edition (NEPSY-II; which assesses a wide range of neuropsychological domains including attention and executive function, language, memory and learning, sensorimotor, social perception, and visuospatial processing), and the Behavior Rating Inventory of Executive Function - Preschool Version (BRIEF-P; which focuses on assessing executive functions, involved in goal-directed behavior in young children ages 2–5 years 11 months). The authors found that higher first-trimester 25(OH)D concentrations were positively associated with verbal development and visuomotor precision. In contrast, third-trimester concentrations were related to visuomotor precision and executive function. The robustness of these findings is strengthened by the careful adjustment for potential confounders, including maternal BMI, diet, lifestyle, and sociodemographic variables.

Importantly, these results build on an earlier study from the same group. In that report, maternal vitamin D status was evaluated in relation to infant neurodevelopment at 40 days postpartum using the Bayley Scales of Infant Development-III.^6^ The study demonstrated that deficient first-trimester 25(OH)D concentrations (<30 nmol/L) predicted poorer performance in infant cognitive and language skills, and third-trimester deficiency (<20 nmol/L) was linked to lower motor outcomes. Together, the two studies from the ECLIPSES cohort illustrate a consistent and concerning pattern: maternal vitamin D deficiency during pregnancy—particularly in the third trimester—is associated with suboptimal neurodevelopmental outcomes, spanning from infancy to preschool age.

The findings are notable not only for their clinical relevance but also for their methodological rigor. The authors thoughtfully considered environmental and behavioral covariates and used well-established neurodevelopmental tools to assess outcomes. Their stratification by trimester allowed for a nuanced interpretation of how maternal vitamin D status may influence different domains of neurodevelopment at varying points during gestation. Importantly, only 16.6% of women had 25(OH)D concentrations >50 nmol/L in the first trimester—a threshold commonly used to define sufficiency—highlighting a persistent gap in maternal nutrition that may have long-term developmental consequences.

These results align with and extend the findings of our group, as reported in Rodgers et al.^8^ In that *post hoc* analysis of a randomized trial, we demonstrated associations between maternal vitamin D supplementation during pregnancy, offspring vitamin D status at testing, and child neurodevelopmental outcomes. Higher 25(OH)D concentrations in children at the time of testing were linked to better performance on the Brigance developmental assessment. Our study also suggested a potential interaction between vitamin D binding protein (VDBP) genotype and developmental outcomes, a variable not assessed in the ECLIPSES cohort but worth exploring in future analyses, particularly given its relevance to vitamin D transport and bioavailability.^9^

Together, these studies underscore the multifactorial nature of vitamin D’s role in early life development. While the timing and magnitude of vitamin D exposure appear critical, so too may be postnatal vitamin D status and individual genetic variability in vitamin D metabolism. The absence of child 25(OH)D measurements in the ECLIPSES study is a limitation that should be addressed in future research. Longitudinal assessment of both maternal and child vitamin D status may provide a more complete picture of the windows of vulnerability and opportunity for intervention.

The findings also prompt a reconsideration of recent clinical practice guidelines. In 2024, the Endocrine Society issued an updated position recommending against the routine measurement of vitamin D status in the general population—including most pregnant women—and advised supplementation with 400 IU/day, except in cases of known deficiency.^10^ While the guidance acknowledges 5000 IU/day as a safe upper limit, this blanket approach may be overly simplistic. Given the substantial variability in individual 25(OH)D concentrations driven by factors such as sun exposure, skin pigmentation, geographic latitude, dietary intake, and genetic differences (e.g., vitamin D binding protein [VDBP] genotype), a one-size-fits-all strategy is unlikely to achieve sufficiency in all pregnant individuals. Personalized assessment and dosing may be necessary to support optimal maternal and fetal outcomes.

In the context of the ECLIPSES and Rodgers et al. findings, it is reasonable to argue that personalized assessment of vitamin D status—particularly during pregnancy—has merit. Not measuring maternal 25(OH)D concentration is analogous to treating hypothyroidism without checking thyroid hormone levels. Monitoring vitamin D status enables tailored supplementation strategies, which may be especially important during critical windows of fetal brain development.

Future studies should include repeated measures of 25(OH)D throughout pregnancy and early childhood, consider genetic polymorphisms that influence vitamin D metabolism, and explore the potential threshold effects that may differ by neurodevelopmental domain. Additional randomized trials with neurodevelopmental outcomes as a primary endpoint would help confirm causality and inform public health recommendations.

In conclusion, the study by Valera-Gran et al. adds to a growing consensus that adequate maternal vitamin D status during pregnancy plays a crucial role in offspring neurocognitive development. These findings, when considered alongside prior work, support the need for continued investigation and potentially revised guidelines that recognize the complexity of vitamin D physiology and its long-term implications for child health.
